# Growth hormone deficiency: craniofacial characteristics and changes after growth hormone therapy - a systematic review

**DOI:** 10.1007/s12020-026-04763-9

**Published:** 2026-09-09

**Authors:** Panagiotis Pallis, Ioannis Padouvas, Evangelos Theodoros Markos, Ioanna Pouliezou, Iosif Sifakakis

**Affiliations:** 1 School of Dentistry, National and Kapodistrian University of Athens, 2 Thivon Str, Athens, 11527 Greece; 2 Department of Orthodontics, School of Dentistry, National and Kapodistrian University of Athens, 2 Thivon Str, Athens, 11527 Greece

**Keywords:** Growth hormone deficiency, Craniofacial morphology, Growth hormone therapy, Dental maturation, Dentofacial growth

## Abstract

**Background:**

Growth hormone deficiency (GHD) is among the most common pituitary hormone disorders in children, causing impaired growth in children and may also affect craniofacial and dental development. Growth hormone therapy (GHT) is the main course of treatment. Despite the high prevalence of GHD, the craniofacial phenotype associated with GHD and the changes observed during therapy have been only partially reviewed in the current literature.

**Purpose:**

This systematic review aimed to compile evidence from January 2000 to April 2026 regarding the craniofacial features of children and adolescents with non-syndromic GHD, as well as the craniofacial changes observed during GHT.

**Methods:**

PubMed, Google Scholar and Scopus were searched for studies published from 2000 to April 2026 that assessed craniofacial or dental changes in non-syndromic GHD patients undergoing GHT. Two reviewers screened the studies and assessed risk of bias using the AXIS tool and the Newcastle-Ottawa Scale. Owing to methodological and clinical heterogeneity, a meta-analysis was not performed.

**Results:**

A total of 621 records were identified; after removing duplicates and applying screening criteria, 10 studies were included. Baseline data generally showed that children with GHD had smaller cranial base and jaw dimensions, retrognathic maxillary and mandibular positions, increased facial convexity, and delayed dental development or eruption compared to healthy peers. Studies on GHT mostly reported craniofacial growth approaching reference values, especially in the maxilla, mandibular ramus, and facial height. Nonetheless, the evidence was limited by small sample sizes, diverse outcome measures, mixed control groups, and generally moderate risk of bias.

**Conclusion:**

Available observational evidence indicates that non-syndromic GHD is associated with a characteristic pattern of craniofacial underdevelopment and delayed dentofacial maturation, whereas GHT may partially normalize several craniofacial parameters. These findings should be interpreted with caution because all included studies were observational and clinically diverse.

**Supplementary information:**

The online version contains supplementary material available at https://doi.org/10.1007/s12020-026-04763-9.

## Introduction

Growth hormone (GH) is a polypeptide protein produced, stored, and released by the anterior pituitary gland in response to hypothalamic growth hormone-releasing hormone (GHRH). Its systemic effects are primarily mediated through insulin-like growth factor 1 (IGF-1), which controls cellular growth, skeletal development, and tissue maturation [1, 2]. Proper GH/IGF-1 signaling is therefore crucial for normal somatic and craniofacial growth [2, 3].

The diagnosis of Growth Hormone Deficiency (GHD) in many countries is established when a severe short stature is observed and the patient’s GH concentration remains below 10 ng/mL after stimulation tests [1, 2]. However, the diagnosis of pediatric GHD is a multistep clinical process that includes longitudinal auxology and growth velocity, clinical findings, biochemical evaluation, pituitary imaging, genetic assessment when indicated, and GH stimulation testing. A peak GH concentration below 10 ng/mL has traditionally been used in some countries, including the United States, but the diagnostic threshold is not universal and varies according to the stimulation test, assay, and local guidance; therefore, a stimulation-test result should not be interpreted in isolation [4, 5].

Idiopathic Short Stature (ISS), by contrast, is a heterogeneous diagnosis of exclusion applied to children with height below 2 standard deviations (SDS) in whom recognized systemic, nutritional, endocrine, chromosomal, or other causes of growth failure have not been identified after appropriate evaluation. Advances in genomic testing show that some children previously classified as having ISS have variants affecting growth-plate or other growth-regulating pathways rather than the GH axis [1, 2, 5, 6].

Growth hormone deficiency (GHD) is among the most common pituitary hormone disorders in children and adolescents [7]. In Europe, prevalence varies from 1 in 1,107 to 1 in 8,646 [7]. For confirmed isolated pediatric GHD, standard therapy is recombinant human growth hormone administered as a daily preparation or long-acting growth hormone (LAGH) administered weekly [8–10].

Recombinant IGF-1 is used primarily for severe primary IGF-1 deficiency, while GnRH analogues are reserved for selected disorders or specific pubertal-management strategies and are not principal treatments for isolated GHD [9]. Studies show that quality of life is similar in children receiving LAGH or rhGH treatment [7]. Recombinant GHT has been successfully used in children with GHD or ISS since 1985 [1, 2]. In children with GHD, rhGH has an immediate and strong impact, improving their height standard deviation score [1, 2]. GH treatment may also be considered for ISS in jurisdictions where it is approved; however, ISS is not equivalent to GHD, and eligibility criteria, dosing, treatment goals, and expected responses differ between the two conditions [1, 2, 5, 9].

Recombinant human GHT has been used in children with GHD since the mid-1980s and remains a cornerstone of management [1, 2, 8]. Beyond improvements in stature, GHT may influence craniofacial growth trajectories, particularly in cartilage-mediated growth sites such as the cranial base synchondroses and the mandibular condyle [3]. These treatment-related changes are of direct interest to orthodontists because they may alter growth patterns, skeletal relationships, and timing of dentofacial interventions.

Growth hormone plays a vital role in bone development, primarily affecting the growth plate, while also aiding in bone remodeling, calcification, and fibroblast growth. According to several studies, a deficiency of growth hormone impacts skeletal growth throughout the entire human body [1–3, 11, 12]. Specifically, in children with GHD, observable features include an immature facial pattern, a retrognathic mandible, a short anterior facial height, and delayed dental eruption [13–15].

Another recent systematic review addressed craniofacial changes in children and adolescents with GHD or ISS receiving GHT, and while its literature search concluded in November 2024, the last included study was in 2017 [12]. Their PECO framework required GH-treated participants and an untreated comparator and therefore focused on treatment-associated changes. The present review addresses a complementary, broader question by synthesizing both (i) the baseline craniofacial and dental phenotype reported in children with non-syndromic GHD, including comparisons with healthy reference groups, and (ii) craniofacial changes reported during GHT. It also updates the search through April 2026. Accordingly, the main contribution of the present review is the integrated, diagnosis-aware synthesis of baseline phenotype and treatment-associated findings rather than a simple extension of the search date.

The present systematic review therefore aimed to synthesize evidence published from January 2000 to April 2026 on the baseline craniofacial characteristics of children and adolescents with non-syndromic GHD and the reported effects of GHT on the craniofacial complex.

## Methods

### Protocol and registration

The review protocol was registered with PROSPERO (CRD420251181114). The review was conducted and reported in accordance with the Preferred Reporting Items for Systematic Reviews and Meta-Analyses (PRISMA) 2020 statement [16].

### Eligibility criteria

The review included observational (cross-sectional and prospective longitudinal/cohort) and interventional studies involving children or adolescents with non-syndromic GHD before and after therapy. Studies were eligible when they evaluated craniofacial morphology, craniofacial growth, dental maturation, tooth eruption, or related dentofacial outcomes, either at baseline or during/after GHT. Studies that included comparison groups with healthy peers or ISS were also eligible, provided that the findings for patients with GHD were presented separately or could be clearly interpreted in relation to GHD.

Exclusion criteria included animal or in vitro studies, syndromic conditions related to growth issues (such as Turner syndrome or Down syndrome), case reports, descriptive papers without analytical outcomes, conference abstracts only, systematic reviews, and opinion articles.

### Search strategy

PubMed, Google Scholar and Scopus were searched for articles published from January 2000 to April 2026. The search strategy was structured according to the PICO framework [17] and combined Medical Subject Headings (MeSH) or keyword synonyms with Boolean operators. The searches integrated terms related to the target populations (e.g., growth hormone deficiency), the intervention (e.g., rhGH, somatropin) and the outcomes of interest (e.g., craniofacial development, cephalometric morphology). The detailed, reproducible search strategy is presented in Supplementary Table S1.

Two reviewers (P.P. and P.I.) independently screened the records for eligibility. Duplicate records were removed with Zotero. Four authors (S.I., P.P., P.I. and M.E.) then assessed titles and abstracts, retrieved full texts for potentially eligible records, and resolved disagreements by discussion.

### Data extraction and risk-of-bias assessment

Four authors (S.I., P.P., P.I. and M.E.) extracted the data into a predefined spreadsheet. The extracted variables included study design, country, participant characteristics, comparator groups, GH exposure or treatment duration, craniofacial and dental outcomes, and the main study findings.

Risk of bias was assessed independently by two reviewers (P.P. and P.I.). Cross-sectional studies were appraised with the AXIS tool [18], whereas prospective longitudinal/cohort studies were appraised with the Newcastle-Ottawa Scale [19]. For the Newcastle-Ottawa Scale, 9/9 stars were interpreted as good (low risk of bias), 7–8/9 stars as satisfactory (moderate risk of bias), 0–6/9 stars as unsatisfactory (substantial risk of bias). For the AXIS tool, the studies were evaluated using a score out of 20, although there is no validated threshold for moderate or high risk of bias. For that reason, there was also a qualitative interpretation of each score [18].

### Data synthesis

Due to increased heterogeneity among the studies (patient sample, GHT options, different durations of treatment, different types of studies), only a descriptive analysis was performed. Since the included studies varied significantly in design, age ranges, comparator groups, treatment durations, and craniofacial outcome measures, statistical pooling was deemed inappropriate. The findings were thus summarized narratively.

## Results

### Study selection

**Fig. 1:**
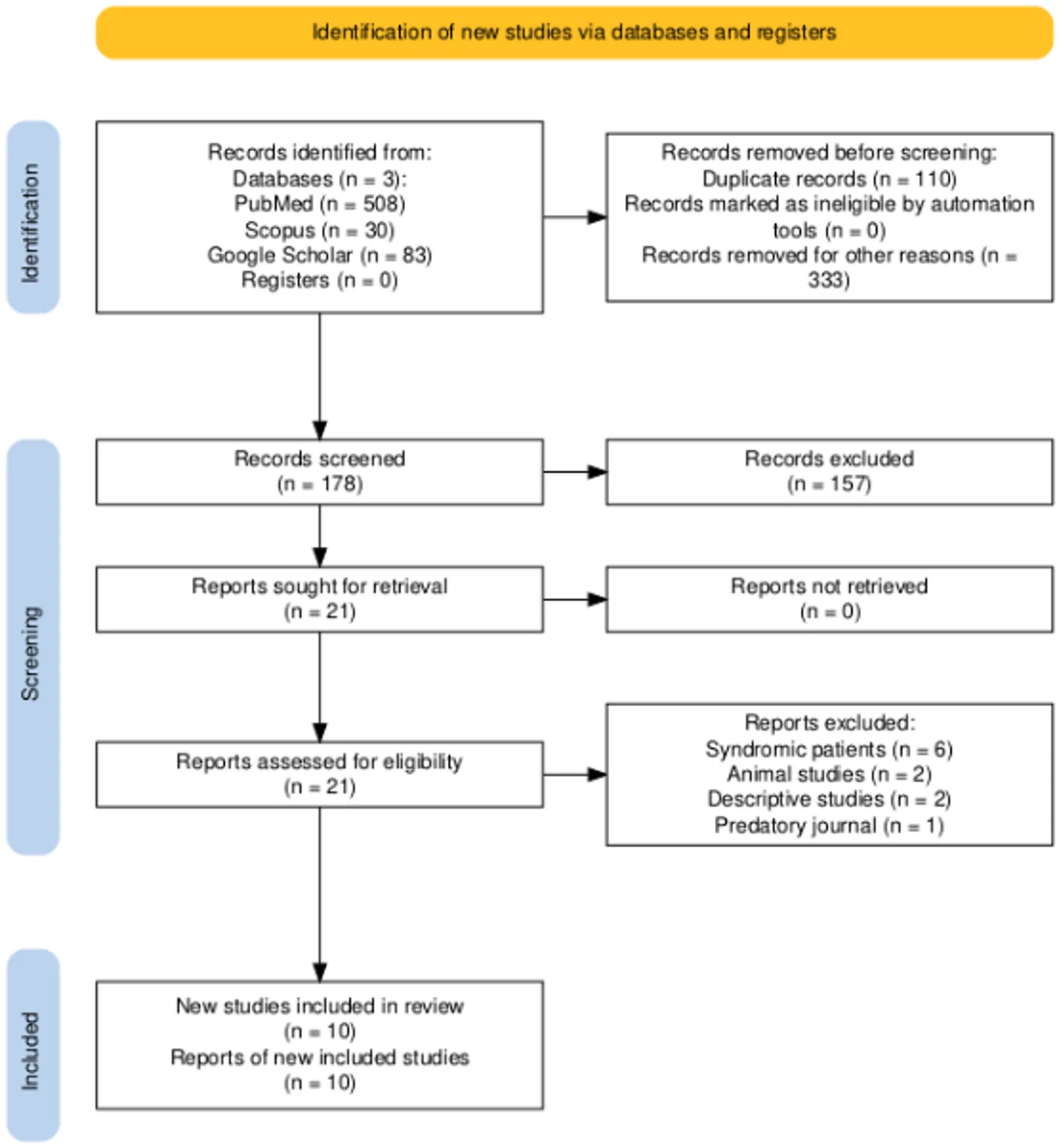
PRISMA2020 flow diagram

The database search yielded 621 records: 508 from PubMed, 83 from Google Scholar, and 30 from Scopus. After removing 110 duplicates, and 333 studies because of irrelevance, 178 records remained for screening. Of these, 157 were excluded after title/abstract or full-text assessment according to the predefined eligibility criteria. Ten studies were ultimately included in the review: seven cross-sectional studies [13–15, 20–23] and three prospective longitudinal/cohort studies [24–26]. Figure 1 illustrates the literature selection process [27].

### Study characteristics

The included studies were conducted in Sweden, Korea, Japan, Mexico, Poland, Romania, Brazil, and the United States. Sample sizes were generally small, ranging from 13 to 110 participants. Most studies used lateral cephalometric radiographs to evaluate craniofacial morphology, while some also assessed dental maturity, tooth eruption, facial morphometric measurements, or head circumference. The characteristics and main results of the individual studies were tabulated as shown in Table 1. The data extracted into the table included: (1) study author and year of publication, (2) intervention, (3) outcomes measured, (4) country of origin, (5) duration and provider of the intervention, (6) number of subjects per group, (7) context of intervention delivery, (8) results, (9) conclusions, and (10) study type.

**Table 1:** Characteristics of the included studies

Study	Design / sample	Comparison	Treatment duration	Results	Main findings	Interpretation / note
Kjellberg et al. (2000) [13]	Cross-sectional baseline; 48 boys with short stature and 109 references	GHD and non-GHD short stature vs. reference subjects	Not applicable	Linear facial measurements were significantly smaller. Showed facial retrognathia and a steep vertical inclination of the mandible. Dental maturity/eruption delayed (1.2 year and 1.3 year, respectively). No significant difference is found between GHD and non-GHD boys.	Smaller facial dimensions, facial retrognathia, and delayed dental maturity and eruption were observed in short-stature boys, including those with GHD.	Supports delayed dentofacial development in this population.
Segal et al. (2004) [23]	Cross-sectional; 52 treated GHD, 12 untreated GHD, 41 relatives	Treated vs. untreated GHD; relatives for morphometric reference	Median 4.3 years	Untreated GHD had smaller face height/width and small head circumference (SDS, − 2.16), but large hands for Height Age (HA) (+ 0.95 SDS). GH-treated subjects showed proportional facial heights, but head circumference increased disproportionately to height (large for stature, *p* = 0.001), correlating significantly with cumulative dose (*r* = 0.55, *P* < 0.0001). Hands/feet were large for stature but proportional to height.	Untreated GHD showed reduced facial height/width; treated children showed proportional facial-height improvement but greater head circumference relative to stature.	Highlights potential heterogeneity of treatment response.
Funatsu et al. (2006) [14]	Cross-sectional; 57 children with GHD	Untreated vs. short-term vs. long-term GHT	> 2 years for long-term group	Long-term therapy group had significantly larger upper facial height (*P* < 0.05), maxillary length (*P* < 0.01), and ramus height (*P* < 0.01) compared to untreated group.	Long-term therapy group showed greater upper facial height, maxillary length, and ramus height than untreated children.	Treatment-related differences were more evident after longer exposure.
Kjellberg and Albertsson Wikland (2007) [24]	Prospective longitudinal; 46 boys	GHD and ISS during GHT vs. healthy references	Mean 6.4 years	Overall enhancement in facial growth. Growth induced a more prognathic pattern and increased anterior rotation of the mandible. Mandibular corpus length and anterior face height exceeded reference norms at the end. No difference in growth response between GHD/ISS groups or low/high dose groups. Earlier start age correlated with larger positive effects.	Overall enhancement of facial growth, more prognathic pattern, and anterior mandibular rotation; about 71% of variables reached or approached normal values.	Earlier treatment start was associated with more favorable changes.
de Faria et al. (2009) [26]	Prospective longitudinal; 20 patients with GHD	Naïve vs. ongoing therapy groups	Mean 3.0 years and 6.5 years	Group 1 (Naive) showed a significant increase in the posterior base of the skull (S-Ar), lower jaw (Co-Gn), and lower third of the face (NS-Me) (*p* < 0.05). Group 2 (Ongoing) showed no significant differences. None developed facial disharmony.	Largest craniofacial changes occurred during the first years of therapy; no facial disharmony reported.	Suggesting early treatment period may be most dynamic.
Salas-Flores et al. (2010) [21]	Cross-sectional; 46 children with GHD (4–18 years)	Treated GHD vs. healthy reference group	Not reported	Boys showed smaller measurements for all facial structures and retrognathic facial type. Girls showed a shortened posterior cranial base (S-Ba 29.14 ± 3.02 mm).	Smaller craniofacial measurements and retrognathic facial pattern; girls showed shortened posterior cranial base.	Supports baseline cephalometric assessment in children with GHD.
Choi et al. (2017) [25]	Prospective longitudinal; 36 children	GHD vs. ISS before/after GHT	2 years	Maxillary length increased beyond norm (> 3 SDS) in boys of both groups (*p* = 0.035). ISS girls showed significantly greater increases in mandibular ramus height (*p* = 0.001), corpus length, and total mandibular length (> 3–6 SDS) compared to GHD girls. ISS girls also showed greater total anterior facial height (> 7–11 SDS).	Maxillary length increased in both groups; girls with ISS showed greater mandibular and facial height increases than girls with GHD.	Indicates diagnosis-specific differences in growth response.
Partyka et al. (2018) [22]	Cross-sectional comparative; 110 children with SH and 41 controls	At treatment initiation vs. during GHT vs. healthy controls	2–3 years in treated subgroup	Skeletal age (19.83 months mean difference, *p* = 0.000) and dental age (8.26 months mean difference, *p* = 0.000) were significantly delayed in SH children compared to birth age. In the course of treatment, no significant difference was found between birth age and dental age (*p* = 0.645). Longer GHT reduces disparities in jaw size.	Skeletal and dental age were delayed at baseline; disparity in dental age was smaller in children already receiving therapy.	Suggests for improved dental maturation during treatment.
Preda et al. (2019) [15]	Cross-sectional; 13 children with isolated GHD	Single-group comparison with reference values	Not clearly reported	Recognition of posterior cranial base, total cranial base, anteroposterior maxillary length, SNA, SNB, ANB, jaw length, and retrognathism is crucial for orthodontic planning in IGHD patients. Study limited by small sample size.	Reduced cranial base and jaw dimensions; low SNA and SNB values indicated maxillary and mandibular retrusion.	Small sample; provides detailed cephalometric description of baseline morphology.
Kim et al. (2021) [20]	Cross-sectional comparative; 95 children	GHD vs. ISS vs. normal controls	Not applicable	Both short-statured groups (SS-HD and SS-I) had smaller cranial base length and facial retrognathia compared to NC. SS-HD group showed significant differences in more variables (7 linear, 6 angular) compared to NC.	GHD group showed reduced cranial base dimensions and more marked retrognathic craniofacial pattern than controls.	Baseline craniofacial phenotype of GHD appeared more pronounced than that of ISS.

### Risk of bias in studies

Among the three longitudinal/cohort studies, two were rated as satisfactory (8/9) and one as unsatisfactory (6/9) on the Newcastle-Ottawa Scale. Among the seven cross-sectional studies, five showed moderate methodological concerns and two showed substantial concerns based on AXIS appraisal. The most frequent limitations across the evidence base were lack of sample-size justification, incomplete reporting on non-responders, limited control of confounding, and imperfect representativeness of the study samples (Tables 2 and 3, Supplementary tables S2 and S3). One study with 14/20 was funded by a source that could affect the interpretation of the results [13], and another study with 15/20 did not mention ethical approval for participants, nor did it discuss the study’s limitations [22].

**Table 2:** Risk-of-bias summary for cross-sectional studies

Study	Tool	Score	Overall appraisal	Main concerns
Kjellberg et al. (2000) [13]	AXIS	14/20	Substantial concerns	Selection bias concerns; no sample-size justification; no information on non-responders.
Segal et al. (2004) [23]	AXIS	16/20	Moderate concerns	No sample-size justification; no information on non-responders.
Funatsu et al. (2006) [14]	AXIS	16/20	Moderate concerns	No sample-size justification; no information on non-responders.
Salas-Flores et al. (2010) [21]	AXIS	17/20	Moderate concerns	No sample-size justification; no information on non-responders.
Partyka et al. (2018) [22]	AXIS	15/20	Substantial concerns	No sample-size justification; no information on non-responders; ethics reporting and limitations incompletely described.
Preda et al. (2019) [15]	AXIS	17/20	Moderate concerns	No sample-size justification; no information on non-responders.
Kim et al. (2021) [20]	AXIS	17/20	Moderate concerns	No sample-size justification; no information on non-responders.

**Table 3:** Risk-of-bias summary for longitudinal/cohort studies

Study	Tool	Score	Overall appraisal	Main concerns
Kjellberg and Albertsson Wikland (2007) [24]	Newcastle-Ottawa Scale	8/9	Satisfactory	Generally robust follow-up; some residual risk of confounding.
de Faria et al. (2009) [26]	Newcastle-Ottawa Scale	6/9	Unsatisfactory	Lack of non-exposed cohort and limited comparability/control of confounding.
Choi et al. (2017) [25]	Newcastle-Ottawa Scale	8/9	Satisfactory	Good outcome assessment and follow-up; some residual confounding remains possible.

### Results of synthesis

#### Baseline craniofacial characteristics in GHD

Across the baseline and comparative studies, children with GHD generally demonstrated smaller craniofacial dimensions than healthy controls, with shorter cranial base measurements, reduced jaw length, and a more retrognathic facial profile [13, 15, 20, 21]. Kim et al. reported that children with GHD (SS-HD) differed significantly from normal controls in a wider range of linear and angular cephalometric variables than children with ISS (SS-I) did, with a more pronounced retrognathic pattern and reduced cranial base dimensions, indicating that the degree of craniofacial deviation from normal was more pronounced in GHD than in ISS [20]. However, not all findings from short-stature cohorts can be attributed specifically to GHD. Kjellberg et al. included 20 boys with GHD and 28 boys with non-GHD short stature, found no significant between-group differences, and therefore pooled both groups as ‘short-statured boys’. Short-statured boys had smaller linear facial measurements [except for the mandibular corpus (Gn-tGo) and the anterior cranial base (S-N)], retrognathia of the mandible (*p* < 0.000) and the maxilla (*p* = 0.004), as well as delayed dental maturity and tooth eruption relative to reference subjects. The smaller facial dimensions, retrognathia, and delayed dental maturation reported in that study should consequently be interpreted as features of short stature broadly rather than as GHD-specific findings [13]. In Choi et al., the baseline Class II difference was observed among girls with ISS compared with girls with GHD and reference participants; this finding does not support a GHD-specific Class II pattern [25].

Salas-Flores et al. also observed craniofacial deficiencies in 46 children with GHD (ages: 4–18), with male subjects showing smaller measurements across facial structures and female subjects exhibiting a shortened posterior cranial base (S-Ba: 29.14 ± 3.02 mm) [21]. Preda et al. described an immature craniofacial pattern characterized by reduced cranial base length, shorter jaws, and retruded maxillary and mandibular positions, reflected in low SNA and SNB values [15]. Collectively, these studies indicate that untreated or baseline GHD is commonly associated with underdeveloped craniofacial structures rather than with an isolated local skeletal deficit.

Dental development appeared to follow the same delayed pattern. Kjellberg et al. reported delayed tooth eruption and dental maturity [13], while Partyka et al. found significantly delayed skeletal and dental age in children with somatotropin hypopituitarism at treatment initiation [22].

#### Reported craniofacial changes during GHT

The longitudinal and treatment-comparison studies generally reported craniofacial growth toward reference values during GHT, particularly in structures influenced by cartilage-mediated growth. Funatsu et al. observed greater upper facial height, maxillary length and mandibular ramus height in children receiving long-term therapy compared with untreated children, whereas short-term therapy was associated with fewer differences [14].

Kjellberg & Wikland (2007) followed boys with GHD or ISS until the end of the growth period and found that GHT was associated with an overall enhancement of facial growth, a more prognathic growth pattern, and anterior rotation of the mandible, with approximately 71% of measured variables reaching or approaching normal values; however, repeated measures ANOVA did not reveal any significant differences between boys with GHD and boys with ISS [24]. Earlier initiation of treatment was associated with more favorable changes in several variables [24]. De Faria et al. similarly reported that the greatest craniofacial changes occurred during the first years of therapy and did not observe facial disharmony during follow-up [26].

Some studies have also reported treatment-related changes in dental maturation. Partyka et al. found that the difference between dental age and chronological age was reduced in children who had been receiving GHT for 2–3 years [22]. Additionally, Choi et al. reported diagnosis-related differences within the female subgroup: girls with ISS had larger treatment-associated increases in mandibular ramus height, mandibular length, and anterior facial height than girls with GHD. Among boys, maxillary length increased beyond reference values in both diagnostic groups. These findings indicate between-diagnosis heterogeneity, particularly among girls, rather than a general sex effect [25]. Segal et al. observed that normalization of facial height was positively correlated with the duration of therapy and cumulative GH dose [23]. Evaluation of facial photographs confirmed that no patient developed acromegalic facial disharmony during treatment [26].

## Discussion

### Summary of evidence

Growth hormone is a complex peptide hormone produced in the pituitary gland of the brain that regulates normal human development, especially of the craniofacial complex. Any disruption in this finely tuned system can lead to GHD. Untreated children with GHD typically exhibit a distinct craniofacial pattern that negatively affects multiple craniofacial structures [14, 15]. This review indicates that non-syndromic GHD is consistently associated with a recognizable pattern of craniofacial underdevelopment. The most frequently reported features were reduced cranial base and jaw dimensions, maxillary and mandibular retrusion, increased facial convexity, and delayed dentofacial maturation [13, 15, 20–22]. These findings are biologically plausible given the central role of GH and IGF-1 in skeletal growth and maturation [2, 3].

The effect of GHD also influences dental development. Dental age and tooth eruption are significantly delayed in children with GHD compared to their chronological age, due to the direct effects of GH and IGF-1 on the differentiation of tooth tissues [13, 22]. Specifically, GH affects Hertwig’s sheath, thereby influencing root formation and odontogenesis [22]. Additionally, a narrow upper arch and overall reduced size of both the maxilla and mandible often lead to increased dental crowding and a greater need for orthodontic evaluation [13, 22].

GHT significantly accelerates craniofacial growth and dental maturation in children with GHD, leading to a phenomenon called “catch-up growth” [22, 24]. The administration of GH mainly stimulates sites of endochondral ossification, which are cartilage-based growth centers [24]. In the craniofacial complex, this specific function has a profound effect on the spheno-occipital synchondrosis of the cranial base and the condylar cartilage of the mandible [13, 24]. When GH stimulates the stem cells within the mandibular condyle, they proliferate into chondrocytes [24, 26]. This vigorous condylar growth changes the mandible’s growth direction, causing it to rotate forward and downward, with the condyle as the center of rotation. This rotation corrects the lower facial height, the relationship between the maxilla and the mandible, and the angle between the mandibular plane and maxillary plane [3, 13, 24]. It also corrects the lower anterior height, reduces the profile curvature, and improves the sagittal mandibular relationship with the maxilla (SNA, SNB, ANB angles), thereby significantly decreasing the Class II skeletal relationships seen in such patients [13, 14].

The magnitude and pattern of reported changes appeared to vary with treatment duration, age at treatment initiation, and diagnostic group, although the evidence was limited and inconsistent [14, 24–26]. The Choi et al. findings should be interpreted as diagnosis-related differences observed within girls, not as evidence of a general sex-dependent treatment effect [25]. The effect of the GHΤ is heavily affected by the duration of the therapy and the patient’s sex [13, 14]. Studies show that the fastest catch-up growth occurs between the first and second years of treatment [14, 26]. Nevertheless, the normalization of critical vertical dimensions, such as the mandibular ramus height and the length of the maxilla, is usually achieved only after a long-term treatment of more than 2 years [14]. Moreover, the timing of the intervention is crucial for the patient to gain the maximum benefit from the GHT. When started at a younger age, treatment can last longer and offer a significantly better correction of the craniofacial structure [24, 25].

The included treatment studies also suggest that GHΤ is associated with craniofacial growth toward reference values, particularly in the maxilla, mandibular ramus, and vertical facial dimensions [14, 22–26]. However, the available evidence should not be interpreted as proving a direct causal effect of therapy on every craniofacial outcome. All included studies were observational, several had small sample sizes, and many used heterogeneous comparators and outcome definitions. The findings therefore support an association between GHΤ and favorable craniofacial growth patterns rather than a definitive causal estimate.

Although the overall direction of findings suggested improvement toward normative craniofacial growth, the evidence was not entirely uniform. Segal et al. reported a proportional improvement in facial height in treated children, which was positively correlated with the duration of therapy and the cumulative GH dose. However, they also observed excessive head circumference growth in association with cumulative GH exposure [23]. These findings highlight the importance of careful clinical interpretation and vigilant monitoring during treatment.

Nevertheless, a recent systematic review concluded that GHT was not associated with adverse effects on general health or craniofacial growth [7]. The latter review is consistent with the present findings, indicating that GHT improves craniofacial growth toward normal dimensions and proportions. In addition, the current review provides a more comprehensive analysis of pretreatment patient characteristics and further elucidates the biomechanical mechanisms underlying craniofacial correction. Compared with Nascimento et al., which evaluated treatment-associated changes using untreated comparators, the present review additionally synthesizes baseline craniofacial and dental findings from studies using healthy reference groups or reporting GHD-specific morphology. This broader scope provides clinical context for interpreting treatment studies, but it also increases heterogeneity and does not yield stronger causal evidence.

### Clinical relevance

The present updated search captures several recent primary studies, providing a more robust and current analysis. From an orthodontic perspective, knowledge of the craniofacial and dental features reported in children with GHD may inform assessment, monitoring, and the timing of orthodontic care. GHT is prescribed for endocrine indications and should not be interpreted as an orthodontic treatment. Coordination between pediatric endocrinologists and dental or orthodontic clinicians may be useful when treatment overlaps with active craniofacial growth.

### Limitations

The findings of this review should be interpreted in light of several methodological limitations. The search was restricted to three databases and to studies published from 2000 onward. Studying this area is especially challenging due to the need for large sample sizes and long-term patient follow-up. The included evidence base was relatively small and consisted almost entirely of observational studies; well-designed clinical trials are lacking. In addition, the methodological heterogeneity of the available primary studies precluded meta-analysis. Moreover, several included studies combined GHD with ISS or other short-stature groups, and some did not report diagnosis-stratified results. Such findings were treated as evidence of short stature in general and were not specifically attributed to GHD. This limitation reduces the certainty and clinical specificity of inferences concerning the GHD population.

### Future research

Future research would benefit from larger, well-designed prospective studies with standardized cephalometric protocols, clearly defined diagnostic criteria, diagnosis-specific subgroup reporting, and longer follow-up. Such studies would improve the precision and clinical applicability of the evidence for both endocrinologists and orthodontists.

## Conclusion

The available evidence suggests that children with GHD may have reduced craniofacial dimensions, retrognathic skeletal relationships, and delayed dental development compared with healthy peers. However, some frequently cited findings are based on mixed short-stature cohorts and cannot be considered GHD-specific. Observational treatment studies suggest partial movement toward reference values during GHT, especially with longer exposure, but do not establish causality or support GH administration for orthodontic indications. GHT should be prescribed and monitored by pediatric endocrine teams for approved endocrine indications; the orthodontic relevance of these findings lies in assessment, monitoring, and timing of treatment. Larger diagnosis-stratified prospective studies are required.

## Supplementary Information

Below is the link to the electronic supplementary material.

Supplementary Material 1

Supplementary Material 2

Supplementary Material 3

## Data Availability

No datasets were generated or analysed during the current study.
