# Supplementary material for: Growth hormone deficiency: craniofacial characteristics and changes after growth hormone therapy - a systematic review
Source: Endocrine. 2026 Sep 9;91(1):288. doi: 10.1007/s12020-026-04763-9 (PMC13558310; doi:10.1007/s12020-026-04763-9)
Supplement: Supplementary file 1 [file 12020_2026_4763_MOESM1_ESM.pdf]

Article title: Growth hormone deficiency: craniofacial characteristics and changes after growth hormone therapy - a systematic review.

Journal Name: Endocrine

Author names: Panagiotis Pallis, Ioannis Padouvas, Evangelos Theodoros Markos, Ioanna Pouliezou, Iosif Sifakakis

Affiliation of the corresponding author: Department of Orthodontics, School of Dentistry, National and Kapodistrian University of Athens, 2 Thivon Str., 11527 Athens, Greece.

E-mail address of the corresponding author: isifak@dent.uoa.gr

Supplementary Table S1. Electronic Search Strategy

Database	Search Strategy	Results
**PubMed**	("Growth Hormone"[Mesh] OR "growth hormone"[tiab] OR "somatropin"[tiab] OR "rhGH"[tiab]) AND ("Craniofacial Abnormalities"[Mesh] OR "Maxillofacial Development"[Mesh] OR "craniofacial"[tiab] OR "cephalometric"[tiab] OR "maxillofacial"[tiab] OR "mandibular"[tiab] OR "skeletal"[tiab]) AND ("idiopathic short stature"[tiab] OR "growth hormone deficiency"[tiab] OR "short stature"[tiab] OR "ISS"[tiab] OR "GHD"[tiab])	508
**Scopus**	TITLE-ABS-KEY (("growth hormone" OR "somatropin" OR "rhGH") AND ("craniofacial" OR "cephalometric" OR "maxillofacial" OR "mandibular" OR "skeletal") AND ("idiopathic short stature" OR "growth hormone deficiency" OR "short stature" OR "ISS" OR "GHD"))	30
**Google Scholar**	allintitle: "growth hormone" craniofacial OR cephalometric OR mandibular	83
