# Supplementary material for: Growth hormone deficiency: craniofacial characteristics and changes after growth hormone therapy - a systematic review
Source: Endocrine. 2026 Sep 9;91(1):288. doi: 10.1007/s12020-026-04763-9 (PMC13558310; doi:10.1007/s12020-026-04763-9)
Supplement: Supplementary file 2 [file 12020_2026_4763_MOESM2_ESM.pdf]

Article title: Growth hormone deficiency: craniofacial characteristics and changes after growth hormone therapy - a systematic review.

Journal Name: Endocrine

Author names: Panagiotis Pallis, Ioannis Padouvas, Evangelos Theodoros Markos, Ioanna Pouliezou, Iosif Sifakakis

Affiliation of the corresponding author: Department of Orthodontics, School of Dentistry, National and Kapodistrian University of Athens, 2 Thivon Str., 11527 Athens, Greece.

E-mail address of the corresponding author: isifak@dent.uoa.gr

Supplementary Table S2. Detailed Risk of bias for the 7 studies with the AXIS tool.

	**Kjellberg et al. (2000) [13]**	**Segal et al. (2004) [23]**	**Funatsu et al. (2006) [14]**	**Salas-Flores et al. (2010) [21]**	**Partyka et al. (2018) [22]**	**Preda et al. (2019) [15]**	**Kim et al. (2021) [20]**
Were the aims/objectives of the study clear?	Y	Y	Y	Y	Y	Y	Y
Was the study design appropriate for the stated aim(s)?	Y	Y	Y	Y	Y	Y	Y
Was the sample size justified?	N	N	N	N	N	N	N
Was the target/reference population clearly defined? (Is it clear who the research was about?)	Y	Y	Y	Y	Y	Y	Y
Was the sample frame taken from an appropriate population base so that it closely represented the target/reference population under investigation?	N	Y	Y	Y	Y	Y	Y
Was the selection process likely to select subjects/participants that were representative of the target/reference population under investigation?	N	Y	Y	Y	Y	Y	Y

Were measures undertaken to address and categorise non-responders?	N	N	N	N	N	N	N
Were the risk factor and outcome variables measured appropriate to the aims of the study?	Y	Y	Y	Y	Y	Y	Y
Were the risk factor and outcome variables measured correctly using instruments/measurements that had been trialled, piloted or published previously?	Y	Y	Y	Y	Y	Y	Y
Is it clear what was used to determine statistical significance and/or precision estimates? (e.g. p-values, confidence intervals)	Y	Y	Y	Y	Y	Y	Y
Were the methods (including statistical methods) sufficiently described to enable them to be repeated?	Y	Y	Y	Y	Y	Y	Y
Were the basic data adequately described?	Y	Y	Y	Y	Y	Y	Y

Does the response rate raise concerns about non-response bias?	N	N	N	N	N	N	N
If appropriate, was information about non-responders described?	N	N	N	N	N	N	N
Were the results internally consistent?	Y	Y	Y	Y	Y	Y	Y
Were the results presented for all the analyses described in the methods?	Y	Y	Y	Y	Y	Y	Y
Were the authors' discussions and conclusions justified by the results?	Y	Y	Y	Y	Y	Y	Y
Were the limitations of the study discussed?	Y	Y	Y	Y	N	Y	Y
Were there any funding sources or conflicts of interest that may affect the authors' interpretation of the results?	Y	Y	Y	N	N	Y	N
Was ethical approval or consent of participants attained?	Y	Y	Y	Y	DN	Y	Y

	14/20. Unjustified sample size, bias in subject selection, no information about the no responders, funding sources that may affect the interpretation of the results	16/20. Unjustified sample size and no information and measures undertaken about the no responders. Funding source that may affect the interpretation of the results.	16/20. Unjustified sample size and no information and measures undertaken about the no responders. Funding source that may affect the interpretation of the results.	17/20. Unjustified sample size and no information and measures undertaken about the no responders	15/20. Unjustified sample size and no information and measures undertaken about the no responders. Not mentioned if ethical approval of participants was obtained. No discussion about limitations	17/20. Unjustified sample size and no information and measures undertaken about the no responders. Funding source that may affect the interpretation of the results.	17/20. Unjustified sample size and no information and measures undertaken about the no responders
