# Supplementary material for: Growth hormone deficiency: craniofacial characteristics and changes after growth hormone therapy - a systematic review
Source: Endocrine. 2026 Sep 9;91(1):288. doi: 10.1007/s12020-026-04763-9 (PMC13558310; doi:10.1007/s12020-026-04763-9)
Supplement: Supplementary file 3 [file 12020_2026_4763_MOESM3_ESM.pdf]

Article title: Growth hormone deficiency: craniofacial characteristics and changes after growth hormone therapy - a systematic review.

Journal Name: Endocrine

Author names: Panagiotis Pallis, Ioannis Padouvas, Evangelos Theodoros Markos, Ioanna Pouliezou, Iosif Sifakakis

Affiliation of the corresponding author: Department of Orthodontics, School of Dentistry, National and Kapodistrian University of Athens, 2 Thivon Str., 11527 Athens, Greece.

E-mail address of the corresponding author: isifak@dent.uoa.gr

Supplementary Table S3. Detailed Risk of bias for the 3 studies with the Newcastle-Ottawa Quality Assessment Scale

	**Kjellberg et al. (2007) [24]**	**de Faria et al. (2009) [26]**	**Choi et al. (2017) [25]**
Representativeness of the exposed cohort	1	1	1
Selection of the non exposed cohort	1		1
Ascertainment of exposure	1	1	1
Demonstration that outcome of interest was not present at start of study	1	1	1
Comparability of cohorts on the basis of the design or analysis Most important factor (Age) Second most important factor (Sex)	1		1
Assessment of outcome	1	1	1
Was follow-up long enough for outcomes to occur (over 2 years)	1	1	1
Adequacy of follow up of cohorts	1	1	1
**Appraisal Score**	8 out of 9	6 out of 9	8 out of 9
**Quality Category (Risk of Bias)**	Satisfactory	Unsatisfactory	Satisfactory
